# Voice Hygiene

**Published:** 1907-07-06

**Authors:** 


### Laryngology and Rhinology.

### VOICE HYGIENE.

Want of observation of the ordinary rules of health and misdirected energy in using the voice are the chief causes of the breakdown of the untrained vocalist, bringing him to the practitioner for treatment under such names as cold, clergyman's sore-throat, or hysterical aphonia. Such conditions are easily prevented and treated by following rules which are readily understood when the physiology of the voice is analysed. Briefly, voice is the product of a current of air emitted from the chest under the control of the respiratory muscles, and set into vibrations by the movements of the vocal cords. Those vibrations are resonated in the cavities above the glottis, and formed into articulate speech by the movements of the tongue in the mouth and pharynx. The most delicate part of this mechanism is the glottis, and our efforts are directed to preserving it intact for its proper function of causing vibrations in the issuing current of air; when this is damaged, natural efforts at compensation are apt to start a true vicious circle. This is easily recognised when one attempts without training to sing up the scale. The glottis, feeling itself failing, endeavours, by increased muscular effort, to maintain its efficiency till its own intrinsic muscles being used up, it calls in to its aid the constrictors and elevators of the pharynx. But by the use of those muscles "the throat is gripped," and the pharynx diminished in calibre, so that its value as a resonance chamber is lost. The voice is pitched higher, and to maintain this high pitch the expiratory blast must be increased in power, and the glottis is kept constantly contracted. Not only does this require the increased muscular effort which started the vicious circle, but the frequent violent closure of the glottis injures its mucous membrane, hinders the circulation, and sets up a laryngitis, which still further injures the function of the larynx. The final stage is reached when the muscles are completely worn out, and aphonia results.

The least deterioration of the proper function of the larynx having such results, the causes thereof must be investigated and removed. Apart from gross destruction by disease, which is not considered here, the causes are twofold: first, insufficient control of the expiratory current by the respiratory muscles; and, secondly, disease of the cavities above the glottis. To control respiration the ordinary individual uses his glottis in addition to the respiratory muscles; but when, as in vocalists, the glottis is more frequently called upon to exercise its true function, the strain of controlling the respiration also is too great, and the larynx deteriorates. To avoid this the diaphragm must be accustomed to keep complete voluntary control of the respiration. High authorities regard the proper function of the thoracic muscles as merely that of fixing the chest in the full inspiratory position, "the high chest position." The position, however, entails such rigid contraction of the neck muscles and consequent venous engorgement that it is best avoided, and a style of breathing more nearly normal adopted. The spinal column is in truth the only proper *point d'appui* for the mechanism of respiration. All exercises should include some performed lying flat on the back. The exercises should ring the changes on slow deep inspiration and expiration, sudden inspiration and expiration, and graduated expiration. General muscular development must be maintained by ordinary hygienic measures: brisk rubbing after bathing, the use of dumb-bells or Indian clubs, or other athletic exercises adapted to the individual.

In discussing the second cause of deterioration of the larynx—disease of the upper cavities—it would be beyond the scope of this article to include more than such disease as is barely considered a departure from the normal—namely, catarrh of the mucous membrane—rhinitis, naso-pharyngitis, pharyngitis, and stomatitis. Those conditions cause swelling of parts, and so diminish the size and resonating value of the chambers involved; they further give rise to increased secretion, which, gravitating down on to the cords, sets up laryngitis either by infection or by mechanical insult. Further efforts are made to expel the inspissated secretion by violent coughing, so that the glottis is still further injured. The treatment of catarrh depends on the region involved; in the nose and naso-pharynx a nasal lotion of sod. bicarb., borax, sacch. alb., of each gr. iij.; acid carbol., gr. j.; aq. dist., ℥j., will lessen secretion and turbinal hypertrophy, and adenoids may require operative treatment. Pharyngitis *per se* merely requires the use of any simple throat lozenge, but the underlying cause must be discovered and treated, notably the excessive use of alcohol and tobacco; the ingestion of highly seasoned food and hot drinks; the various disorders of digestion, especially constipation; rhinitis, tonsillitis, and, above all, stomatitis. The oral cavity has been left to the last, but only to emphasise its importance in all disorders of the pharynx and larynx. The sequels of dental stomatitis are well known. The teeth must be carefully examined, tooth-plates being always removed for this purpose.

Finally, it should be mentioned that careful and distinct articulation takes the strain so much off the larynx that all regular voice users should practise it.

